# Stereotactic Thermal Ablation of Liver Tumors: 3D Planning, Multiple Needle Approach, and Intraprocedural Image Fusion Are the Key to Success—A Narrative Review

**DOI:** 10.3390/biology10070644

**Published:** 2021-07-10

**Authors:** Gregor Laimer, Peter Schullian, Reto Bale

**Affiliations:** Interventionelle Onkologie—Mikroinvasive Therapie (SIP), Medizinische Universität Innsbruck, Anichstr. 35, A-6020 Innsbruck, Austria; gregor.laimer@i-med.ac.at (G.L.); peter.schullian@i-med.ac.at (P.S.)

**Keywords:** thermal ablation, navigation, image fusion, stereotaxy, reliability

## Abstract

**Simple Summary:**

Thermal ablation is a minimally invasive, potentially curative approach for the treatment of primary and secondary liver tumors. Different technologies are available, with radiofrequency ablation (RFA) and microwave ablation (MWA) being the most widely used. Both methods induce an irreverible destruction of the tumor tissue by applying thermal energy. Conventionally, a single ablation probe is placed using ultrasound (US) or computed tomography (CT). Using these standard guidance techniques the creation of necrosis zones to treat large tumors (i.e., >2–3 cm) is hampered by technical limitations. These limitations can be overcome by stereotactic thermal ablation: a multiple needle approach with 3D treatment planning and precise stereotactic needle placement combined with immediate verification of treatment success by means of image fusion. The stereotactic multi-needle technique allows for local curative treatment of multiple and large tumors within a single session. Ultimately, due to the application of these sophisticated tools and advanced techniques, a local curative treatment option can be offered to a higher number of patients suffering of primary and secondary liver tumors. This article provides an overview of the current stereotactic techniques for thermal ablation and summarizes the available clinical evidence for this approach.

**Abstract:**

Thermal ablation is an emerging, potentially curative approach in treating primary and metastatic liver cancer. Different technologies are available, with radiofrequency ablation (RFA) and microwave ablation (MWA) being the most widely used. Regardless of the technique, destruction of the entire tumor, including an adequate safety margin, is key. In conventional single-probe US- or CT-guided thermal ablation, the creation of such large necrosis zones is often hampered by technical limitations, especially for large tumors (i.e., >2–3 cm). These limitations have been overcome by stereotactic RFA (SRFA): a multiple needle approach with 3D treatment planning and precise stereotactic needle placement combined with intraprocedural image fusion of pre- and post-interventional CT scans for verification of treatment success. With these sophisticated tools and advanced techniques, the spectrum of locally curable liver malignancies can be dramatically increased. Thus, we strongly believe that stereotactic thermal ablation can become a cornerstone in the treatment of liver malignancies, as it offers all the benefits of a minimally invasive method while providing oncological outcomes comparable to surgery. This article provides an overview of current stereotactic techniques for thermal ablation, summarizes the available clinical evidence for this approach, and discusses its advantages.

## 1. Introduction

Percutaneous thermal ablation is an emerging alternative, potentially curative approach in the treatment of primary and metastatic liver malignancies. As a minimally invasive approach, it is tissue sparing, it rarely evokes major complications, and treatment-associated mortality is exceedingly low [1]. Different technologies are available, such as radiofrequency ablation (RFA), microwave ablation (MWA), laser ablation, or cryoablation. Of those, RFA and MWA are by far the most known and widely used modalities for the treatment of liver tumors.

The ultimate goal of any curative local treatment of liver cancer is eradication of the whole tumor, avoiding local tumor progression (LTP) and, thus, increasing patient survival. To achieve this goal for thermal ablation, it is key to cover the whole tumor with necrosis zone, including an adequate safety margin [2]. This safety margin, defined as the shortest distance between tumor border and margin of the necrosis zone, is a critical determinant for successful ablations [3]. In conventional, US- or CT-guided single-probe thermal ablation the creation of such large necrosis zones is often hampered by technical limitations, especially for large tumors (i.e., >2–3 cm). Therefore, advances in techniques and technologies are needed as the demand for minimally invasive methods continues to grow.

The aim of this narrative review is to provide a comprehensive overview of current stereotactic techniques for thermal ablation, to summarize the available clinical evidence for this approach, and to discuss its advantages compared to conventional guidance techniques.

Unfortunately, a search in the database PubMed (U.S. National Library of Medicine, http://www.ncbi.nlm.nih.gov/pubmed, accessed on 10 May 2021) with the search criteria: “Liver” AND “ablation” AND “stereotactic” yielded only a limited number of publications on stereotactic thermal ablation, most of them from the center in Innsbruck and some from two other centers: the University of Bern and Stockholm. Therefore, this review focuses mainly on the technique of stereotactic thermal ablation adopted by the center in Innsbruck, the importance of intraprocedural image fusion, briefly reviews the similar but different stereotactic approach of the Bern/Stockholm group, and discusses the advantages in the treatment of liver tumors using stereotactic thermal ablation in general.

## 2. Conventional Thermal Ablation—The Limitations

### 2.1. Image Guidance

Ultrasound (US) is the most widely used image guidance for thermal ablation. Conventional B-mode US seems to be the ideal targeting modality considering it is fast, inexpensive, and allows real-time visualization of the probe during placement. However, this technique is strongly dependent on the skills of the performing interventionalist. Additionally, it is limited by the sonographic window and mainly by inconspicuous tumors. As many as 20–25% of small hepatocellular carcinomas (HCCs) detected on pre-interventional cross-sectional imaging are not visible on preprocedural US, with subphrenic locations and the presence of liver cirrhosis being independent predictors of invisibility [4,5]. Furthermore, probe repositioning during the ablation process is hampered by the development of gas bubbles and many tumor locations require double-angled approaches. Because of these limitations, up to 45% of planned US-guided ablations may not be feasible [6]. 

CT guidance is another widely used imaging modality for the placement of ablation probes and monitoring of the necrosis zone. In contrast to US, CT enables a 3D view of the tumor and surrounding structures. It is useful for tumors that are not visible on US or located in the hepatic dome, which necessitate a trans-pleural approach [7]. Disadvantages of CT as image guidance include radiation exposure, a limited angle for in-plane ablation probe placement (CT gantry), as well as—in the case of absence of an especially dedicated interventional suite—blockage of the device for diagnostic purposes. Intravenous contrast application is essential for a sufficient soft tissue contrast and for visualization of intrahepatic vessels. Nevertheless, the short time-window after intravenous application of the contrast agent for proper localization, probe placement, and ablation of the liver lesion is additionally hampered by the non-real-time nature of the modality. 

Even though MRI as an image guidance would offer several advantages [8,9], such as higher sensitivity for small lesions, superior visibility of tumor/necrosis zone, or the possibility to monitor the ablation temperature (thermometry), it is rarely used for the ablation of liver tumors. The reasons for this include limited availability of MR scanners and MRI-compatible ablation devices, long procedural times, and high costs. 

### 2.2. Tumor Size—How to Break the Limitation?

Among several variables, such as perfusion and pathological tissue changes, the volume of the necrosis zone mainly depends on the ablation modality. Many institutions prefer MWA to RFA for its ability to achieve larger necrosis zones. In vivo, the short axis diameter of the necrosis zone using straight radiofrequency ablation probes is approximately 1.5–2 cm, whereas it is up to 4 cm for latest generation of MWA probes [10]. Several studies [11,12,13,14,15] reported the peritumoral safety margin as an independent predictor for local tumor progression (LTP). Therefore, a safety margin of 5–10 mm is generally recommended. Thus, the maximum tumor sizes for hepatocellular carcinoma (HCC) and colorectal liver metastases (CRLM) that can be treated from a single-probe position are 3 cm and 2 cm, respectively. In addition, such diameters are only achievable if the probe is perfectly positioned in the center of the lesion. 

However, even with perfect probe placement, tumor size remains the most important and crucial limitation of conventional US- or CT-guided single probe thermal ablation. It is, therefore, not surprising that international guidelines consider thermal ablation only as a valid first-line alternative to surgical resection for singular HCC < 2 cm (“very early” HCC, Barcelona Clinic Liver Cancer 0) [16]. For any other primary or secondary liver tumor, surgical resection is still recommended as the first-line local curative treatment, while thermal ablation is reserved for inoperable liver malignancies [16,17].

Nevertheless, it is possible to treat liver malignancies > 2–3 cm by attaining multiple overlapping necrosis zones in three dimensions, but it remains a very challenging task to achieve by conventional “freehand” US- or CT-guided thermal ablation. This is the main reason for dissatisfying results in large liver tumors. Furthermore, results differ considerably between the interventionalists due to various factors such as levels of skills (hand-eye coordination, three-dimensional imagination) and experience. Thus, a standardized, mostly operator-independent multiple-needle approach, such as SRFA, seems to be the solution, as size-related limitations of thermal ablation are eliminated, allowing to achieve a theoretical ablation area of any size reliably.

## 3. SRFA of Liver Tumors—The Technique

### 3.1. What Is Stereotaxy?

Stereotaxy has been used for decades in neurosurgery for biopsies and tumor treatments and is part of the standard equipment in neurosurgical rooms. Early imitations of frame-based stereotaxy (inflexible surgical access routes, invasive fixation of the frame) have been overcome by the introduction of frameless stereotactic 3D navigation systems in the early 1990s. With these systems it is possible to locate a point within the patient in a 3D coordinate system of a CT-/MRI/PET/SPECT-image in real-time, on the understanding that the registration between both is correct. Modern navigation systems may be used in various body regions due to their flexibility. In addition, the software of most modern navigation systems also allows for planning of multiple needle trajectories in a cartesian coordinate system. After adjustment of aiming devices according to the virtual pre- and intraoperative plan, punctures of almost every anatomical structure are feasible. In passive navigation systems, the aiming device is set manually, but it can also be carried out (semi-) automatically by robot-assisted systems. 

For stereotactic punctures of the liver, it is mandatory to control the respiratory motion of the patient, as it strongly influences accuracy. This can be achieved by temporary endotracheal tube disconnection and complete muscle relaxation during anesthesia [18], by jet ventilation [19,20], or by the THRIVE (Transnasal Humidified Rapid Insufflation Ventilatory Exchange) anesthesia technique [21].

### 3.2. First Stereotactic Thermal Ablation in 2001

The first SRFA of a liver tumor was performed by Bale et al. [22] in 2001, followed by the first stereotactic MWA (SMWA) in 2008, the first stereotactic irreversible electroporation (SIRE) in 2013 and the first stereotactic cryotherapy (SCT) in 2014 by the same group. Til today, more than 1000 patients with more than 4000 tumors have been treated by the team in Innsbruck using stereotactic ablation procedures. All patients and each tumor treated were collected and monitored in a prospective registry, implemented in the hospital information system. 

### 3.3. The Technique for SRFA

At the center of Innsbruck, the intervention itself is performed in a dedicated intervention room with a sliding gantry CT (SOMATOM Sensation Open, Siemens Inc., Erlangen, Germany). The sliding gantry moves on rails between two different rooms, separated by a large mobile lead wall. 

In this article, we provide only a brief description of the technique as a more precise and very detailed one has already been given elsewhere [23,24,25]. The intervention is performed under general anesthesia with deep muscle relaxation. Respiratory triggering is achieved by temporary disconnection of the endotracheal tube. At first, a dual-phase contrast enhanced planning CT (SOMATOM Sensation Open, Siemens Inc.) with 3 mm slice thickness is obtained with the patient already immobilized on the CT table by means of a vacuum mattress. The data are then transferred to the optical based 3D navigation system (S8, Medtronic Inc., Boulder, CO, USA). Multiple trajectories are planned with the 3D navigation system software on multiplanar reformatted images to cover the entire tumor volume with an appropriate peritumoral safety margin. If required, especially for non-visible lesions in the planning CT, it is possible to fuse pre-interventional CT/MRI/PET/SPECT data with the intraprocedural CT dataset [26]. Following registration, accuracy check, and sterile draping, the ATLAS aiming device (Interventional Systems Inc., Kitzbühel, Austria) is adjusted using the 3D navigation system. Then, 15G/17.2 cm coaxial needles (Bard Inc., Covington, GA, USA) are sequentially advanced through the locked aiming device to the preplanned target point. For verification of correct needle placement, a non-enhanced CT scan is obtained and superimposed onto the planning CT. After a 16G biopsy is taken through the coaxial needles, up to three Cool-tip RF-electrodes (Medtronic Inc., Boulder, CO, USA) at a time are introduced through the coaxial needles for serial tumor ablation. The ablation itself is performed with a unipolar ablation device with a switching controller. Needle track ablation is performed during every probe repositioning and during final probe removal to prevent bleeding and potential tumor seeding. A dual phase contrast enhanced CT scan is then acquired to assess complications and the ablation result. As a final step, the post-interventional CT scan is superimposed onto the pre-interventional CT scan to assess the peritumoral safety margin in three dimensions. In case of incomplete ablation (residual tumor; lack of sufficient safety margin), the intervention may be continued in the same session by stereotactic placement of additional coaxial needles with subsequent ablation. 

**Figure 1:**
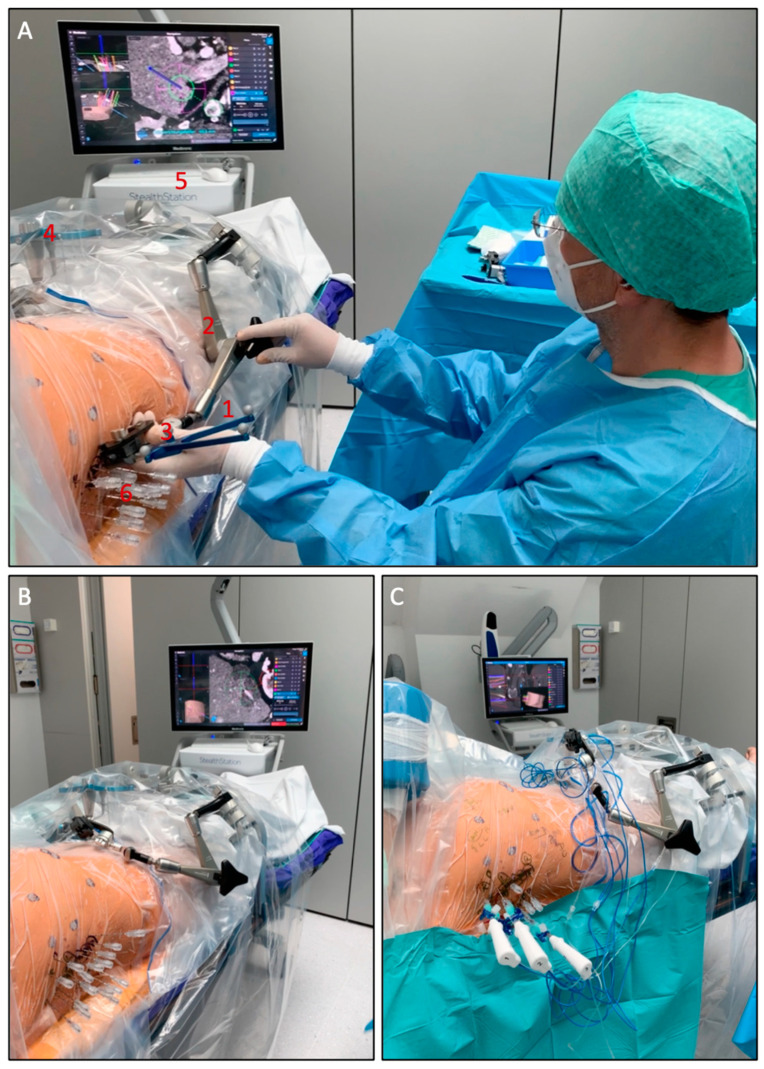
Setup of the SRFA procedure. (**A**) The probe (1) of the navigation system is inserted into the ATLAS aiming device (2). The reflective markers (3) on the probe and the reference frame (4) are tracked by the camera of the navigation system (5). After manual alignment of the aiming device with the virtual trajectory and consecutive removal of the probe, the interventionalist introduces several coaxial needles (6) through the locked aiming device to the preplanned target point. (**B**) Placed coaxial needles in final position. (**C**) After verification of correct needle placement with a non-enhanced control CT scan and image fusion with the planning-CT up to three RF-electrodes at a time are introduced through the coaxial needles for serial tumor ablation.

The setup of the SRFA procedure is shown in Figure 1.

### 3.4. Important Features and Advantages of SRFA

**Figure 2:**
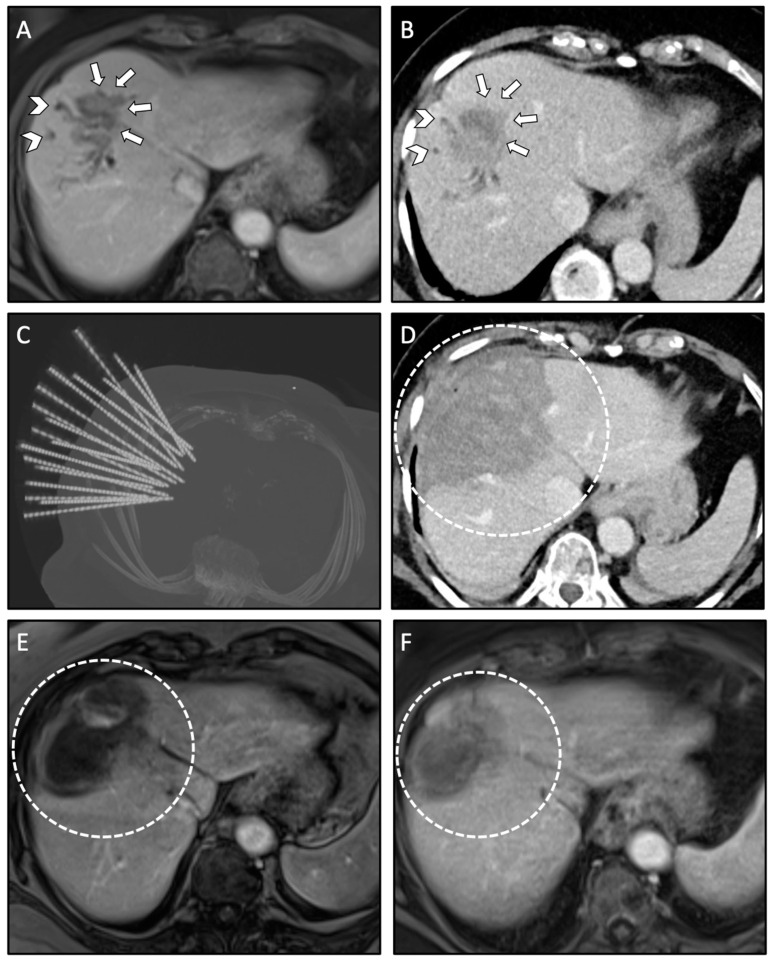
Case of a 76-year-old female with local recurrence of CRLM after conventional US- guided RFA treated by anatomical ablation of liver segment VIII using 18 coaxial needles for SRFA. (**A**,**B**) Pre-interventional MRI scan (**A**) and planning CT scan (**B**) of the SRFA procedure with 45 mm recurrence of the CRLM (white arrows) and consecutive dilated intrahepatic bile ducts in liver segment VIII indicating cholestasis (white arrowhead). (**C**) Maximum Intensity Projection (MIP) of the non-enhanced control CT scan showing the position of 18 coaxial needles. (**D**) Final control CT scan after anatomical ablation with complete necrosis of liver segment VIII. (**E**,**F**) Follow-up MRI scan at 3 months (**E**) and 21 months (**F**) after SRFA, showing no evidence of LTP with shrinking necrosis zones (white dashed circle).

Using coaxial needles as guidance for the ablation probes has several advantages: the coaxial needles (placed before ablating) remain in the planned position relative to the tumor even after tissue shrinkage; large ablation areas can be achieved by placement of multiple coaxial needles. Coaxial needles are less space occupying as compared to ablation probes. This is especially relevant for heavy patients with limited space in the CT gantry; it is financially sustainable, as only up to three RFA probes are required for a feasible serial ablation. Moreover, the use of coaxial needles allows to perform a biopsy of each tumor prior to the ablation. In addition, the use of coaxial needles allows for simultaneous treatment of multiple lesions (up to 24 liver metastases reported by the team in Innsbruck) and very large lesions (up to 18 cm in diameter reported by the team in Innsbruck) within one session. An example with multiple coaxial needles for SRFA is shown in Figure 2.

Respiratory triggering is of utmost importance for SRFA, due to considerable differences in the position of the liver caused by respiratory motion. Thus, it is mandatory that each image acquisition and the needle placement are performed in the same respiratory phase in order to guarantee an identical spatial position of the liver. This respiratory triggering is achieved by temporary endotracheal tube disconnection and complete muscle relaxation during anesthesia. Its safety was evaluated in 26 patients and revealed an overall mean respiratory motion error for internal targets of 1.98 ± 0.93 mm (range, 0.44–4.02 mm) [18]. 

Targeting accuracy is another essential factor for a successful stereotactic approach. The targeting accuracy for stereotactic needle placement in the liver was evaluated in 20 patients with 35 lesions: the mean (±SD) lateral error of 145 placed needles was 3.6 ± 2.5 mm at the tip [27]. 

With proper mentoring, the technique can be learned in a few months. In fact, a study on reliability of SRFA revealed no significant differences between the performance of an interventional oncologist with >10 years of experience, and a young trainee with only two months of SRFA training [28]. The study evaluated technique effectiveness, morbidity, mortality, hospital stay, and LTP in 90 patients treated with SRFA for 72 primary and 105 secondary liver malignancies with a mean size of 2.9 cm (range, 0.5–11 cm). No significant difference between the operators for any of the variables was observed. 

### 3.5. Similar Approaches Elsewhere?

Currently, to our knowledge, the institution in Innsbruck is the only one performing SRFA for liver lesions. Nevertheless, a similar approach using microwave ablation (MWA) as ablation modality was adopted by the University of Bern and Stockholm with a similar planning, navigation, and image fusion software for stereotactic placement of the probes. In contrast to the technique adopted by the Innsbruck group, the probes are directly inserted without the use of coaxial needles. Furthermore, sterile draping is performed before image acquisition and sterile reflective skin markers are used for patient tracking. 

The Bern group reported treatment of 301 primary and secondary liver tumors in 191 ablation sessions with a mean diameter of 1.5 cm using stereotactic MWA [29]. The mean targeting positioning error per probe was 2.9 ± 2.3 mm. The LTP within six months was 22% (49/227), and 35% of the lesions were successfully re-ablated. Lesion size >30 mm and targeting position error > 5 mm were significant influence factors for LTP, while a challenging location had no significant influence on LTP nor on targeting accuracy. The same group published two other studies using stereotactic MWA with remarkable results, one on their initial experiences with non-colorectal liver metastases (NCRLM) [30] and one with hepatocellular carcinoma [31]. Of 40 non-colorectal liver metastases in 23 patients with a median size of 1.4 cm only 4 (10%) showed LTP after a median follow-up of 15 months. One patient (4%) had a major complication, and the median disease-free survival (DFS) and overall survival (OS) were 7 and 18 months, respectively. Of 174 HCCs in 88 patients with a median size of 1.6 cm, only 11 (6.3%) of the lesions recurred on the ablation site after a median follow-up of 17.5 months. Close proximity to major vessels significantly correlated with LTP. In another study [32], the same group assessed the value of MRI/CT image fusion in stereotactic MWA, presenting a safe treatment option for patients with non-detectable malignant liver lesions on contrast-enhanced CT planning scans. Of 24 CT ‘invisible’ lesions, a total of 22 lesions were successfully ablated within the first session, and two were successfully re-ablated afterwards.

In a first study [33], the Stockholm group assessed safety and feasibility of a multiple MWA strategy in patients with non-resectable CRLM. Of 20 ablations, 7 were performed using stereotactic CT navigation. The authors called for further development of this promising technique to enable multiple ablation strategies with minimal surgical access. In another retrospective study [19], the group assessed the accuracy and procedural safety of stereotactic CT-guided percutaneous MWA of liver tumors using high-frequency jet ventilation. Twenty consecutive patients with malignant liver lesions non-eligible for surgical resection or US-guided ablation were included in the study. The mean size of 25 lesions in 17 patients was 14.9 ± 5.9 mm. The antennae of the MWA probe were placed with a mean lateral error of 4.0 ± 2.5 mm, a depth error of 3.4 ± 3.2 mm, and a total error of 5.8 ± 3.2 mm. No major complications occurred. The authors concluded that percutaneous MWA performed with CT-guided stereotactic navigation is technically feasible and suitable for safe treatments, as it provides sufficient accuracy and requires almost no repositioning of the antenna.

### 3.6. Ablation Modality for the Stereotactic Approach: RFA or MWA?

A recent review [2] has clearly highlighted the many advantages of MWA over RFA in conventional thermal ablation, claiming that MWA should be considered the technique of choice for tumors ≥ 3 cm in diameter or close to large vessels. However, if used in a stereotactic setting with coaxial needles, most of the advantages of MWA over RFA (larger necrosis zone; simultaneous ablation) are relativized or even compensated. For this reason, even though the first stereotactic MWA (SMWA) was performed by the Innsbruck group in 2008 using the identical stereotactic setup, they almost exclusively rely on RFA when treating liver lesions. The reasons for this are the following: first, RFA probes are financially more sustainable compared to MWA probes, and since the interventions are performed in a public hospital, a focus on cost-effectiveness is very important. Second, in the case of multiple needle usage, having a smaller thermosphere (short ablation zone diameter RFA: approximately 1.5 cm; MWA: 3–4 cm) allows for a more precise tailoring of the ablation area and consequently less risk of sacrificing healthy tissue or destruction of delicate structures. This is particularly important when a tissue-sparing approach is needed, such as when treating lesions in infants [34] or in organs with a small functional reserve. Third, coaxial needles are ideal for the treatment of multiple lesions within one session, as described above. Unfortunately, the use of coaxial needles for the MWA approach is problematic (or may even be precluded) due to the larger diameters of the MWA probes (up to 13 gauge) as compared to straight RFA probes (17 gauge).

## 4. Image Fusion and Safety Margin—Key to Complete Ablation

As already mentioned above, several studies [11,12,13,14,15] reported the peritumoral safety margin as an independent predictor for local tumor progression (LTP) after thermal ablation of liver malignancies. Safety margins of >5 mm for HCCs and >10 mm for CRLMs were recommended. These observations are further supported by a group of international experts who similarly recommend a margin of >10 mm for CRLMs [35].

The crucial impact of the safety margin on LTP after thermal ablation of liver malignancies was also confirmed by recent data of the Innsbruck group. In an initial study [12], technique efficacy of SRFA was evaluated in 110 patients with 176 HCCs. The safety margin was assessed with a commercially available, rigid imaging registration software and proved to be the only independent predictor for LTP. For each millimeter increase of the margin, a 30% relative risk reduction for LTP was observed. Furthermore, none of the lesions ablated with a safety margin of >5 mm showed LTP in subsequent follow-up CT and MRI scans. 

Another study [13] assessed the safety margin after SRFA of 76 CRLMs in 45 patients, also revealing it as the only significant independent predictor for LTP. In addition, different volumes and percentages for several safety margins (1–10 mm) were calculated using a non-rigid imaging registration software. A circumscribed (100%) safety margin of 3 mm, or at least 90% of a 6-mm circumscribed 3D safety margin, were associated with complete response after a mean follow-up of 36.1 ± 18.5 months. Notably, both studies showed very low overall percentages of LTP with 5.7% for HCCs (10/176) and 11.8% for CRLMs (9/76).

### 4.1. How to Assess the Safety Margin?

Several different approaches for the assessment of the safety margin have been proposed by the scientific community [11,12,13,14,36], including different rigid and non-rigid registration tools for image fusion of pre- and post-interventional CT and MRI scans. Furthermore, Kaye et al. [37] demonstrated in a recent study higher LTP discrimination power of a volumetric 3D assessment of the safety margin compared to a manual 2D approach. 

Nevertheless, most interventionalists neither use a 3D nor a 2D assessment, but rather rely on the conventional approach by visual inspection alone. This conventional approach consists in side-by-side juxtaposition of pre- and post-interventional CT scans, thus containing many possible sources of error such as differences in body position, respiratory motion, or liver deformation (in case of large necrosis zones). Therefore, the evaluation of the technical success after thermal ablation of liver malignancies by visual inspection alone can be very challenging. In fact, in a recent study [38], even experienced interventional radiologists showed major difficulties assessing the technical efficacy of thermal ablation by visual inspection alone. In this study, peri-interventional CT scans of nine patients with HCC referred to SRFA were presented to 38 interventional radiologists from 14 countries. The radiologists had to judge, by conventional side-by-side juxtaposing of images, whether complete ablation (i.e., technical success and technique efficacy) was achieved. Overall, 3.97 ± 1.27 out of 9 (44.1%) cases per radiologist were misjudged. A total of 18/38 (47.4%) of the study participants had considerable experience in percutaneous tumor ablation with >50 interventions performed. Nevertheless, this expertise had no significant influence on the results, with almost identical percentages of misjudged cases. 

### 4.2. Safety Margin Assessment in SRFA

**Figure 3:**
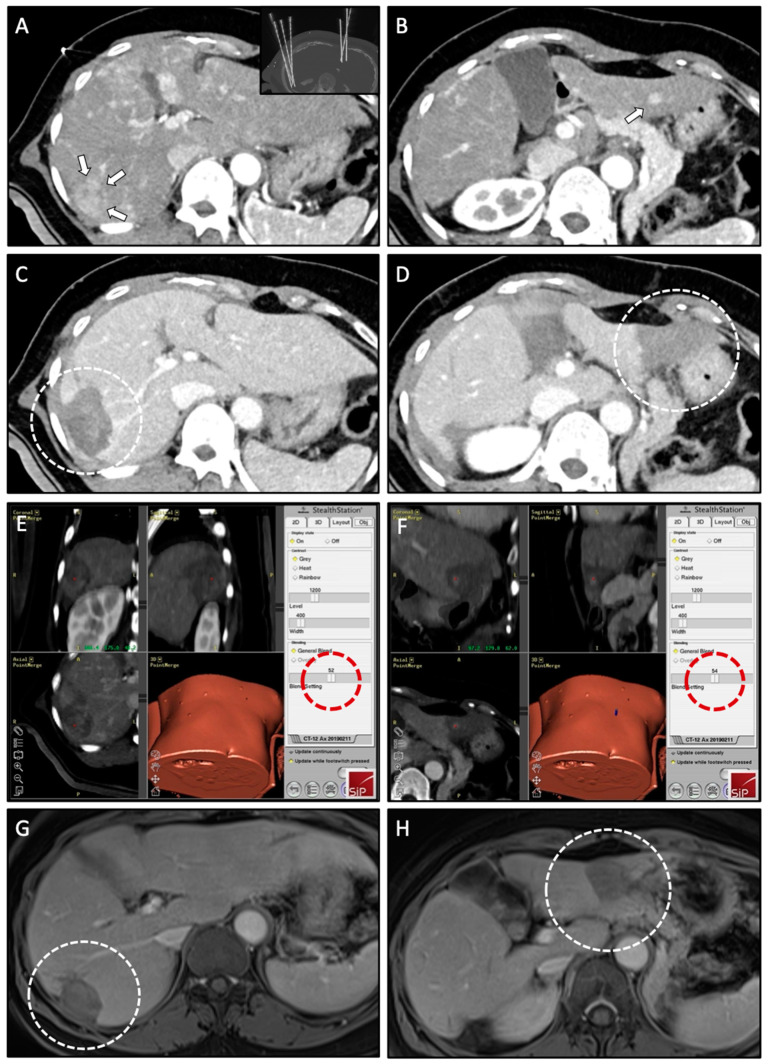
Case of a 57-year-old male with simultaneous treatment of two HCCs (right and left liver lobe) using SRFA. (**A**,**B**) Planning CT scan in arterial phase of the SRFA procedure with a 30-mm HCC nodule in liver segment VI (white arrows) and a 9-mm HCC nodule in liver segment III (white arrow); Maximum Intensity Projection (MIP) of the non-enhanced control CT scan showing the position of seven coaxial needles in the upper right corner of (**A**). (**C**,**D**) Control CT scan after ablation with corresponding necrosis zones in liver segment VI and III (white dashed circles). (**E**,**F**) Three-dimensional views of the intraprocedural image fusion of the contrast-enhanced planning and final control CT scan using the rigid-registration tool from the navigation system with the possibility to switch between images using the blending function (red dashed circle); verification of complete necrosis including a sufficient safety margin. (**G**,**H**) Follow-up MRI scan 18 months after SRFA, showing no evidence of LTP with shrinking necrosis zones (white dashed circle).

As part of the SRFA procedure, an intraprocedural image fusion of pre- and post-ablation contrast-enhanced CT scans (planning/control CT scan as part of the procedure) is performed using a rigid registration tool implemented in the navigation software with final manual adjustment by using anatomical structures. It is an integral and mandatory component of the SRFA workflow (Figure 3) for intraprocedural evaluation of the technical success. In case of incomplete ablation (residual tumor; lack of sufficient safety margin), the intervention may be continued in the same session.

### 4.3. Rigid vs. Non-Rigid Registration Tools

Image registration is the process of determining the geometric transformation that relates identical (anatomic) points in two image series: a moving dataset and a stationary source dataset. Restricting the registration of two images to simple rigid transformations is often met with remaining uncertainties due to the deformable nature of soft tissue [39]. This is especially applicable for the liver, which is sensitive to various body motions, including breathing. However, due to the application of general anesthesia and respiratory triggering during the SRFA workflow, the pre- and post-interventional CT scans are both obtained with the patient (and the respiration depending organs) in an identical position. Elimination of these soft tissue deformations minimizes the inherent limitations of rigid registration tools and facilitates precise registration of pre- and post-interventional CT scans. Several rigid registration tools are already implemented in many diagnostic image-viewing programs and may, therefore, be used for image fusion of pre- and post-interventional CT scans and the assessment of the safety margin, if reasonable considering the limitations. 

However, in cases when the planning CT scan and the control CT scan are obtained in different body and breathing positions, soft tissue deformations enforce the use of non-rigid registration methods. Complex algorithms, which can deform liver parenchyma by referring to intrahepatic landmarks such as vessels, can overcome the limitations of rigid registration tools. Such a fully automatic non rigid image fusion software is already available and has been applied in two recent studies. One assessed the safety margin in HCC patients after conventional US-guided RFA [36] and another in CRLM patients after SRFA [13].

## 5. Results after SRFA—Is It Worth the Effort?

### 5.1. Treatment Efficacy by Histopathological Evaluation

A recent study [40] evaluated the effectiveness of SRFA for HCC in 97 patients for bridging to liver transplantation. The effectiveness was assessed by histopathological examination of the explanted liver, with 183/188 (97.3%) nodules showing complete histopathological response. Tumor size had no significant influence on the results. In fact, complete tumor cell death was achieved in 50/52 (96.2%) nodules ≥3 cm.

### 5.2. Complications after SRFA: A 15-Year Experience

One retrospective study [41] assessed the frequency of major complications after SRFA and putative predictors of adverse events using simple and multivariable logistic regression. From July 2003 to December 2018, a total of 793 patients (median, 65.0 years (range, 0.3–88), 241 women) were treated in 1235 SRFA sessions for 2475 primary and metastatic liver tumors. The median tumor size was 3.0 cm (range, 0.5–18 cm).

Thirty-day mortality after SRFA was 0.5% (6/1235). The major complication rate was 7.4% (91/1235) and decreased significantly from 11.5% (36/314) before January 2011 to 6.0% (55/921) thereafter. Of the major complications, 50.5% (46/91) were successfully treated in the same anesthetic session (angiographic coiling for hemorrhage/chest tube insertion for pneumothorax). Independent predictors for major complications were a history of bile duct surgery/intervention, number of coaxial needles, and location of tumors in segment IVa or VIII following multivariable logistic regression analysis. The number of tumors, tumor size, and location close to the diaphragm, tumor conglomerate, and segment VII were other significant predictors using simple logistic regression.

### 5.3. SRFA for Different Tumor Entities

#### 5.3.1. Colorectal Liver Metastases (CRLM)

Long-term results of SRFA for CRLM were evaluated by an early retrospective analysis of 189 CRLMs in 63 patients from 2005 to 2011 [25]. The median OS was 33.2 months after an average follow-up of 25 months, and was significantly longer for patients with resectable vs. non-resectable lesions (27 vs. 58 months, *p* = 0.002). The OS rates for patients with resectable lesions at 1, 3, and 5 years were 92%, 66%, and 48%, respectively. Overall, LTP occurred in 31/189 (16%) of the lesions. Tumor size had no significant influence on LTP rate, OS, and DFS.

#### 5.3.2. Breast Cancer Liver Metastases (BCLM)

In a recently published study [42], we evaluated the outcomes after SRFA of 110 BCLMs in 42 female patients. A total of 107/110 BCLMs were successfully ablated at initial SRFA (97.3% primary technical efficacy rate), and 1 of 3 tumors was successfully re-treated in a second session (98.2% secondary technical efficacy rate). No procedure related deaths were observed. A total of 4/48 (8.3%) Grade 1 and 1/48 (2.1%) Grade 2 complications, according to the CIRSE Classification System for Complications, were observed. LTP occurred in 8/110 (7.3%) tumors. OS rates for 1, 3, and 5 years from the date of the first SRFA were 84.1%, 49.3%, and 20.8%, respectively. Median OS was 32.3 months. Age of >60 years and extrahepatic disease (excluding bone only metastases) were significant predictors of worse OS. Size and number of metastases, hormone receptor status, and time onset did not significantly affect OS after initial SRFA.

#### 5.3.3. Melanoma Liver Metastases (MLM)

From 2005 to 2013, 75 MLMs in 20 patients were treated with SRFA in 34 sessions [43]. On average, two lesions (range, 1–14) per patient were ablated, with a mean size of 1.7 cm (range, 0.5–14.5 cm). A total of eight lesions were larger than 3 cm (10.7%). There were no procedure-related deaths, and all major complications (*n* = 3) could be easily treated by pleural drainages. The primary and secondary technical success rates were 89.3% and 93.3%, respectively. The LTP rate was 13.3% (10/75). Of those recurrent tumors, 4/10 were successfully re-treated. The median OS from the date of the first SRFA was 19.3 months with OS rates at 1, 3, and 5 years of 64%, 41%, and 17%, respectively.

#### 5.3.4. Intrahepatic Cholangiocellular Carcinoma (ICC)

In 11 inoperable patients with 52 ICCs referred to SRFA from 2004 to 2010, an estimated median OS of 60 months was observed [44]. Median follow-up time was 35 months. The primary and secondary technical success rates were 92% and 98%, respectively. The lesions had a mean diameter of 3 cm (range, 0.5–10 cm). LTP occurred in 3/36 (8%) tumors.

### 5.4. SRFA in Challenging Tumor Locations

#### 5.4.1. Hepatic Dome Lesions

A recent paper [45] evaluated the results after SRFA of 238 tumors (82 HCCs, 6 ICCs, and 89 metastases) in 177 patients located in the hepatic dome. The results were compared with 177 patients randomly selected from an existing database for propensity score matching. Median tumor size was 2.2 cm (range, 0.5–10 cm). Primary technical success rate was 97.5% (232/238), with five tumors successfully re-treated (secondary technical efficacy rate of 99.6%). LTP was observed in 21/238 (8.8%) tumors. The major complication rate was 10.7% (22/204); of those, 55% (12/22) were successfully treated by the interventional radiologist in the same anesthesia session. No significant differences in adverse events or disease control rates were observed between subdiaphragmatic tumors and matched controls.

#### 5.4.2. Subcardiac Hepatocellular Carcinoma

In a similar study [46], the safety and efficacy of SRFA of 114 subcardiac HCCs in 79 patients were evaluated. The results were compared with a randomly selected control group of 79 patients with 242 HCCs referred to SRFA in other (non-subcardiac) locations with propensity score matching. The median tumor size was 2.5 cm (range, 0.5–9.5 cm) and LTP occurred in 8/104 (7.0%) cases. Major complication and perioperative mortality rates were 7.7% (8/104) and 1% (1/104), respectively. The OS rates at 1, 3, and 5 years from the date of the first SRFA with single subcardiac HCCs were 92%, 77%, and 65%, respectively. The median OS was 90.6 months. No significant differences between the subcardiac and control group were observed in terms of local tumor control, safety, OS, and DFS.

#### 5.4.3. Caudate Lobe Lesions

Another study [47] evaluated HCCs in the caudate lobe. Of 24 nodules in 20 patients located in the caudate lobe, only 1 (4.2%) showed LTP in follow-ups. The median OS was 51.3 months. The 1-, 2-, and 5-year OS rates were 95%, 59%, and 44%, respectively.

### 5.5. SRFA in Difficult Situations

#### 5.5.1. CT ‘Invisible’ Lesions

In a recently published study [26] in 60 patients 199 lesions (29 HCC; 170 metastases from other origins) which were not visible in CT were treated by SRFA using image fusion with pre-interventional MRI scans. The results were compared with 60 patients randomly selected using nearest neighbor propensity score matching (‘control group’). The major complication rate was 8.7% (6/69 ablations). Primary technical efficacy rate (i.e., successful initial ablation) was 96.6% (28/29) for HCC and 97.9% (166/170) for metastatic disease. LTP occurred in 1/29 (3.5%) HCCs and in 6/170 (4.0%) metastases. The LTP rate of metastasis in the control group was significantly higher (p = 0.007). For OS and DFS rates, no differences between both groups were observed. Metastases that disappear after systemic chemotherapy are difficult to localize during liver resection. These so-called “vanishing metastases” can easily be located by fusion of MRI datasets prior to systemic treatment to the planning CT and subsequently treated by thermal ablation.

#### 5.5.2. Octogenarians

One study [48] assessed the results of 36 patients > 80 years referred to SRFA for 70 liver tumors in 46 ablation sessions. The primary technical efficacy rate was 97% (68/70). LTP was detected in 5/70 nodules (7.1%). The major complication rate (> Clavien Dindo Grade III) was 6.5% (3/46). The OS rates at 1, 3, and 5 years from the date of the first SRFA were 84.6, 50.5, and 37.9%, respectively, for HCC patients and 87.5% and 52.5% at 1 and 3 years, respectively, for CRC patients. There were no significant differences in terms of technical efficacy, LTP, major complications, OS, and DFS compared to the control group (younger patients were randomly selected from an existing database).

### 5.6. SRFA in Very Large or Multiple Tumors

#### 5.6.1. Tumors > 8 cm

From 2005 to 2018, 34 consecutive patients with 41 primary and metastatic liver tumors with a median size of 9.0 cm (range, 8.0–18.0 cm) were referred to SRFA with curative intent [49]. The primary technical success rate was 80.5% (33/41), and four tumors required repeat ablation (secondary technical efficacy rate 90.2%). LTP occurred in 4/41 tumors (9.8%). The 30-day perioperative mortality was 2.3% (1/44 ablations), and the major complication rate was 20.5% (9/44 ablations). Of those, 3/9 major complications (pleural effusion, pneumothorax, perihepatic hemorrhage) could easily be treated by the interventional radiologist. The OS rates at 1, 3, and 5 years from the date of the first SRFA were 87.1%, 71.8%, and 62.8%, respectively for patients with HCC and 87.5%, 70.0%, and 70.0%, respectively, for patients with ICC. Patients with secondary liver malignancies had OS rates of 77.8% and 22.2% at 1 and 3 years, respectively.

#### 5.6.2. Patients with Four or More Lesions

Another paper [50] assessed the feasibility, safety, and clinical outcome of simultaneous SRFA of four or more primary and metastatic liver malignancies in 92 patients, 35 with ≥4 HCCs and 57 with ≥4 metastatic liver tumors. The median tumor sizes of 178 HCCs and 371 liver metastases were 2.2 cm (range, 1.0–8.5 cm) and 3.0 cm (range, 0.5–13 cm), respectively. At initial SRFA, 7/35 (20%) patients with HCC and 19/57 (33.3%) patients with metastases had ≥6 tumors. Major complication rates for patients with HCC and metastases were 5.4% (2/35 ablations) and 10% (7/63 ablations), respectively. LTP was observed in 4/178 (2.2%) HCCs and in 17/371 (4.6%) metastases. The median OS was 38.2 months (HCC) and 37.4 months (metastases). The OS rates at 1, 3, and 5 years were 88.0%, 54.0%, and 30.4%, respectively, for patients with HCCs, and 86.1%, 53.1%, and 37.3%, respectively, for patients with metastases.

### 5.7. Stereotactic RFA for Recurrent Liver Lesions after Resection

#### 5.7.1. Recurrent HCCs

From 2006 to 2018, 34 consecutive patients with HCC and previous hepatic resection were treated by SRFA for 140 nodules [51]. The median tumor size was 3.0 cm (range, 0.5–10 cm). LTP occurred in 4/140 tumors (2.9%). The major complication rate was 4.8% (3/60 ablations). No periprocedural deaths occurred. The median OS was 69.1 months and OS rates at 1, 3, and 5 years from the date of the first SRFA were 94.0%, 70.2%, and 53.3%, respectively.

#### 5.7.2. Recurrent CRLMs

In a similar study [52], 64 consecutive patients from 2006 to 2018 with recurrent or new CRLM after previous hepatic resection were treated by SRFA for 217 lesions in 103 ablation sessions. The median tumor size was 2.7 cm (range, 1–7.5 cm). Primary technical efficacy rate was 97.7% (213/217 tumors). Four tumors required repeat ablation (99.5% secondary technical efficacy rate). LTP occurred in 25/217 (11.5%) lesions. The major complication rate was 5.8% (6/103 ablation sessions) and the mortality rate was 1.0% (1/103 ablation sessions). The median OS was 33.1 months and OS rates at 1, 3, and 5 years from the date of the first SRFA were 90.1%, 46.2%, and 34.8%, respectively.

### 5.8. Quality of Life after SRFA

One study [53] evaluated the health-related quality of life (HRQoL) after SRFA of liver tumors. Of 577 patients who underwent SRFA for liver tumors in 892 ablation sessions from 2011 to 2017, 303 patients (52.5%) returned 363 completed HRQoL questionnaires once after the ablation (50 patients with multiple questionnaires, due to multiple ablation sessions). If necessary, a total of 355/363 (97.8%) patients indicated willingness to undergo repeat SRFA with little to no fear in 292 (80.7%) patients. Among patients with multiple therapies, SRFA was rated by 132/147 (89.8%) as the preferred re-treatment, hepatic resection by 4/147 (2.7%), and chemotherapy by 11/147 (7.5%).

An overview of recent studies on stereotactic thermal ablation (SRFA and SMWA) is shown in Table 1. This table highlights the excellent oncologic outcomes of stereotactic treatment of liver tumors, as evidenced by consistently low LTP rates, high OS rates, high primary/secondary technical efficacy rates, and very low morbidity and mortality rates.

## 6. Conclusions

Despite improvements of thermal ablation techniques over the past decades, surgical resection is still considered the first-line local curative treatment for most liver tumor entities. In our opinion, the most likely reason, therefore, is that most institutions still rely on single-probe thermal ablation with its inevitable limitations, especially for large tumors (i.e., >3 cm).

As documented in several publications over the last 20 years, the limitations of conventional “freehand” CT- or US-guided thermal ablation (tumor size, tumor location, or number of lesions) can be overcome by SRFA. Using this sophisticated planning and guidance technique, very large (>8 cm) and multiple (>6) tumors can be treated within one session. Due to consistently good oncological outcomes with very low overall LTP rates, high primary and secondary technical efficacy rates, as well as low mortality and morbidity rates, SRFA challenges resection as first-line treatment for liver tumors.

Three-dimensional planning, precise stereotactic placement of multiple needles, and immediate assessment of the safety margin have proven to be the key to success. Each of these components of the SRFA workflow is essential and mandatory for a successful ablation. Three-dimensional planning and stereotactic needle placement allow for precise (multiple) needle placement that can be performed out of plane, improving ablation efficacy. A recent study [54] comparing the primary technical efficacy of CT-navigated stereotactic guidance and manual guidance for percutaneous MWA of liver malignancies was significantly in favor of stereotactic guidance. Assessment of the peritumoral safety margin by image fusion is an integral part of the SRFA workflow and is used as an intraprocedural tool to evaluate local treatment success. We believe that this should be mandatory of any thermal ablation procedure.

We are aware that this review is limited by a mostly single-center bias and the retrospective design of the cited studies. However, we hope that this review aids in generating further interest in the technique of stereotactic thermal ablation. It is of utmost importance that this technique becomes adapted by other centers in order to create prospective multicenter data.

## Figures and Tables

**Table 1:** Overview of recent studies using stereotactic thermal ablation (SRFA and SMWA).

	Tumor Entity	Specialties	Tumor Size, cm; Median (Range)	LTP, %	Primary/Secondary Technical Efficacy Rate, %	OS, Months; Mean	OS 1, 3, and 5 Years, %	Major Complications, %	Mortality, %
SRFA Studies									
Bale et al. [40]	HCC	Histopathological evaluation of response (explanted liver)	2.5 (1–8)	2.7	-	114.3 (with LT)	92, 83, 79	4	-
Schullian et al. [41]	HCC, ICC, CRLM, NCRLM	Frequency and risk factors for major complications	3.0 (0.5–18)	8.3	-	50.0	89, 60, 44	7.4	0.5
Bale et al. [25]	CRLM	-	2.0 (0.5–13)	16.0	-	33.2	87, 44, 22	17.0	0
Schullian et al. [42]	BCLM	-	3.0 (0.8–9)	7.3	97.3/98.2	32.3	84, 49, 21	10.4	0
Bale et al. [43]	MLM	-	1.7 (0.5–14.5)	13.3	89.3/93.3	19.3	64, 41, 17	8.8	0
Haidu et al. [44]	ICC	-	3.0 (0.5–10)	8	92/98	60.0	91, 71, -	13	0
Schullian et al. [45]	HCC, ICC, CRLM, NCRLM	Hepatic dome lesions	2.2 (0.5–10)	8.8	97.5/99.6	66.9 (HCC)	87, 76, 58 (HCC)	10.7	0.5
Schullian et al. [46]	HCC	Subcardiac HCC	2.5 (0.5–9.5)	7.0	95.6/99.1	90.6	92, 77, 65	7.7	1.0
Schullian et al. [47]	HCC	Caudate lobe lesions	1.5 (1–8)	4.2	95.8/100	51.3	95, 59, 44	4.3	4.3
Schullian et al. [26]	HCC, CRLM, NCRLM	CT ‘invisible’ lesions	2.9 (0.5–11) HCC; 2.5 (0.8–9) metastases	3.5 (HCC); 4.0 (metastases)	96.6/- (HCC); 97.9/-(metastases)	42.7 (HCC); 46.0 (metastases)	92, 70, 44 (HCC); 95, 53, 37 (metastases)	8.7	0
Schullian et al. [48]	HCC, ICC, CRLM, NCRLM	Octogenarians	2.7 (1.5–9)	7.1	97.0/100	51.5 (HCC)	85, 51, 38 (HCC)	6.5	0
Schullian et al. [49]	HCC, ICC, CRLM, NCRLM	Very large tumors (>8 cm)	9.0 (8.0–18)	9.8	80.5/90.2	95.4 (HCC)	87, 72, 63 (HCC)	20.5	2.3
Schullian et al. [50]	HCC, CRLM, NCRLM	Four or more lesions	2.7 (0.5–13)	3.8	98.6/99.6	38.2 (HCC); 37.4 (metastases)	88, 54, 30 (HCC); 86, 53, 37 (metastases)	8.4	0
Schullian et al. [51]	HCC	Recurrent HCC after hepatic resection	3.0 (0.5–10)	2.9	95.0/97.9	69.1	94, 71, 53	4.8	0
Schullian et al. [52]	CRLM	Recurrent CRLM after hepatic resection	2.7 (1–7.5)	11.5	97.7/99.5	33.1	90, 46, 35	5.8	1.0
SMWA Studies									
Tinguely et al. [29]	HCC, CRLM, NCRLM	-	1.5 (1.1–2.1)	22	96/-	-	-	2	-
Perrodin et al. [30]	NCRLM	-	1.3 (0.6–3.9)	10	-	18	-	12	-
Lachenmayer et al. [31]	HCC	-	1.6 (0.4–4.5)	6.3	87.9/96.3	-	-	5.9	-
Cathomas et al. [32]	HCC; CRLM, ICC	CT ‘invisible’ lesions	1.2 (0.3–2.8)	-	91.7%/100%	-	-	0	0
Engstrand et al. [19]	HCC, CRLM, NCRLM	Use of high-frequency jet ventilation	1.5 ± 0.6 (mean ± SD)	-	-	-	-	0	0

Abbreviations: -, not applicable; HCC, hepatocellular carcinoma; ICC, Intrahepatic cholangiocellular carcinoma; CRLM, colorectal liver metastases; NCRLM, non-colorectal liver metastases; LTP, local tumor progression; LT, liver transplantation; OS, overall survival.

## Data Availability

Not applicable.

## Abbreviations

RFA	radiofrequency ablation
MWA	microwave ablation
LTP	local tumor progression
US	ultrasound
CT	computed tomography
SRFA	stereotactic radiofrequency ablation
HCC	hepatocellular carcinoma
MRI	magnetic resonance imaging
CRLM	colorectal liver metastases
THRIVE	Transnasal Humidified Rapid Insufflation Ventilatory Exchange
PET	Positron emission tomography
SPECT	Single-photon emission computed tomography
NCRLM	non-colorectal liver metastases
DFS	disease-free survival
OS	overall survival
SMWA	stereotactic microwave ablation
BCLM	breast cancer liver metastases
MLM	melanoma liver metastases
ICC	intrahepatic cholangiocellular carcinoma
HRQoL	health-related quality of life
MIP	Maximum Intensity Projection
